# Supplementary figures and images for: Complementary Roles of Wood-Inhabiting Fungi and Bacteria Facilitate Deadwood Decomposition
Source: mSystems. 2021 Jan 12;6(1):e01078-20. doi: 10.1128/mSystems.01078-20 (PMC7901482; doi:10.1128/mSystems.01078-20)

# Metatranscriptome

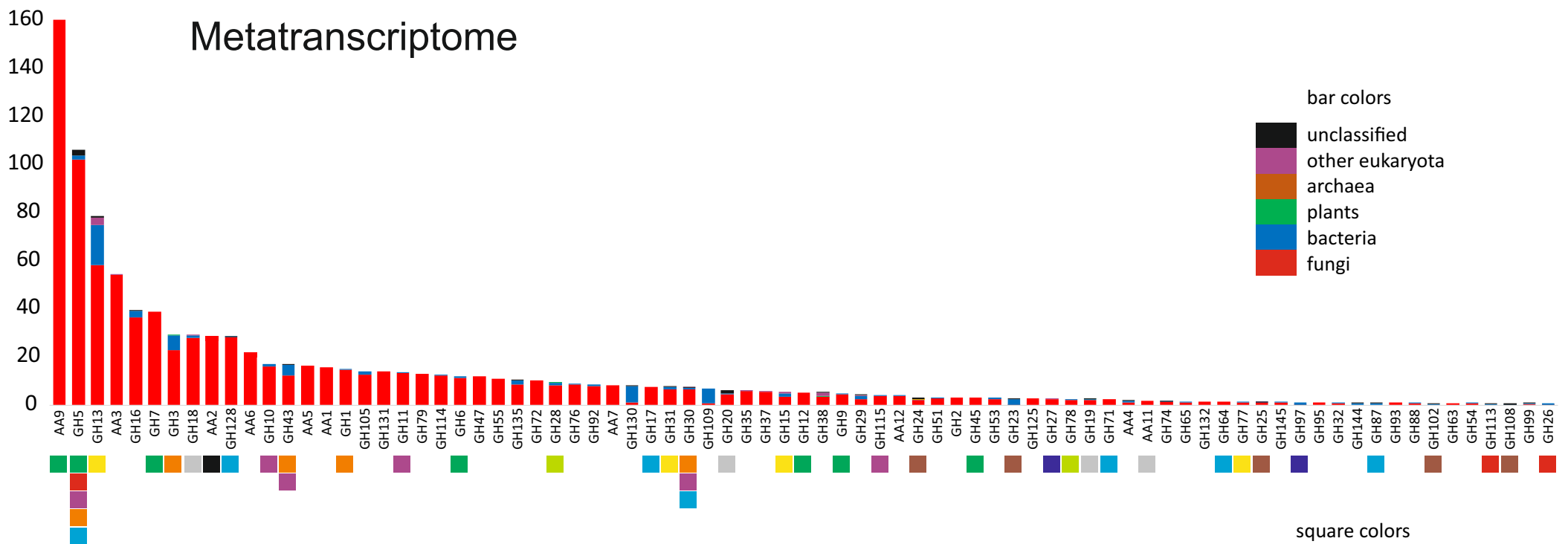

# Metagenome

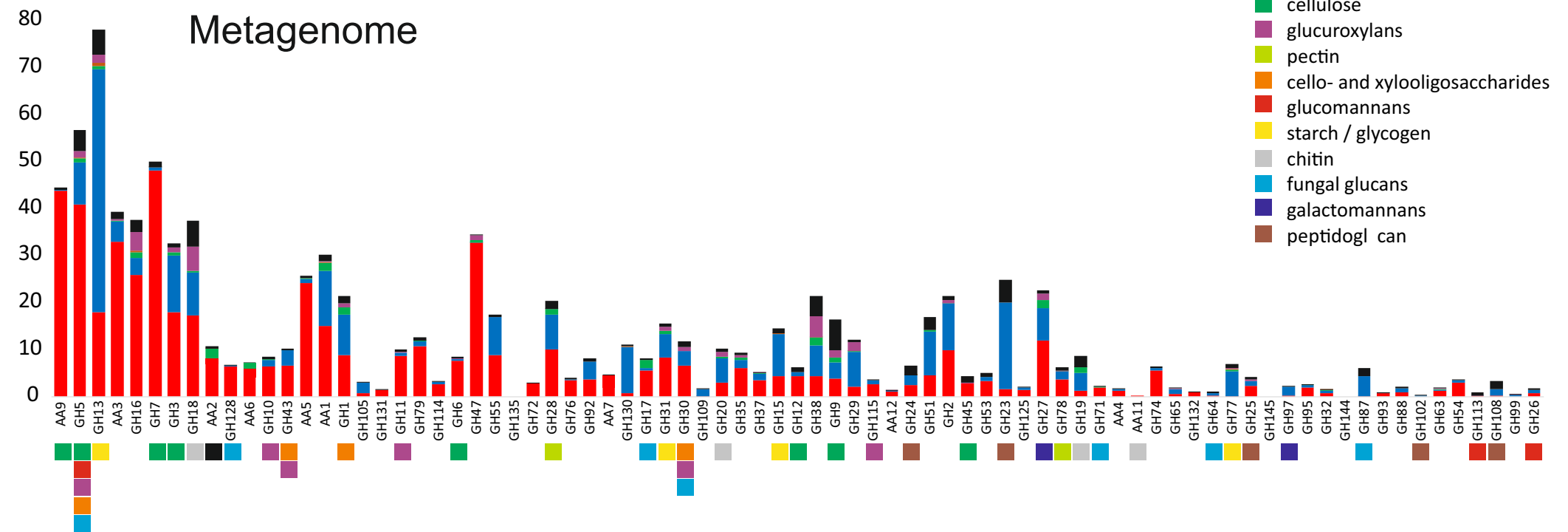

Supplement: FIG S1 [file mSystems.01078-20-sf001.pdf]

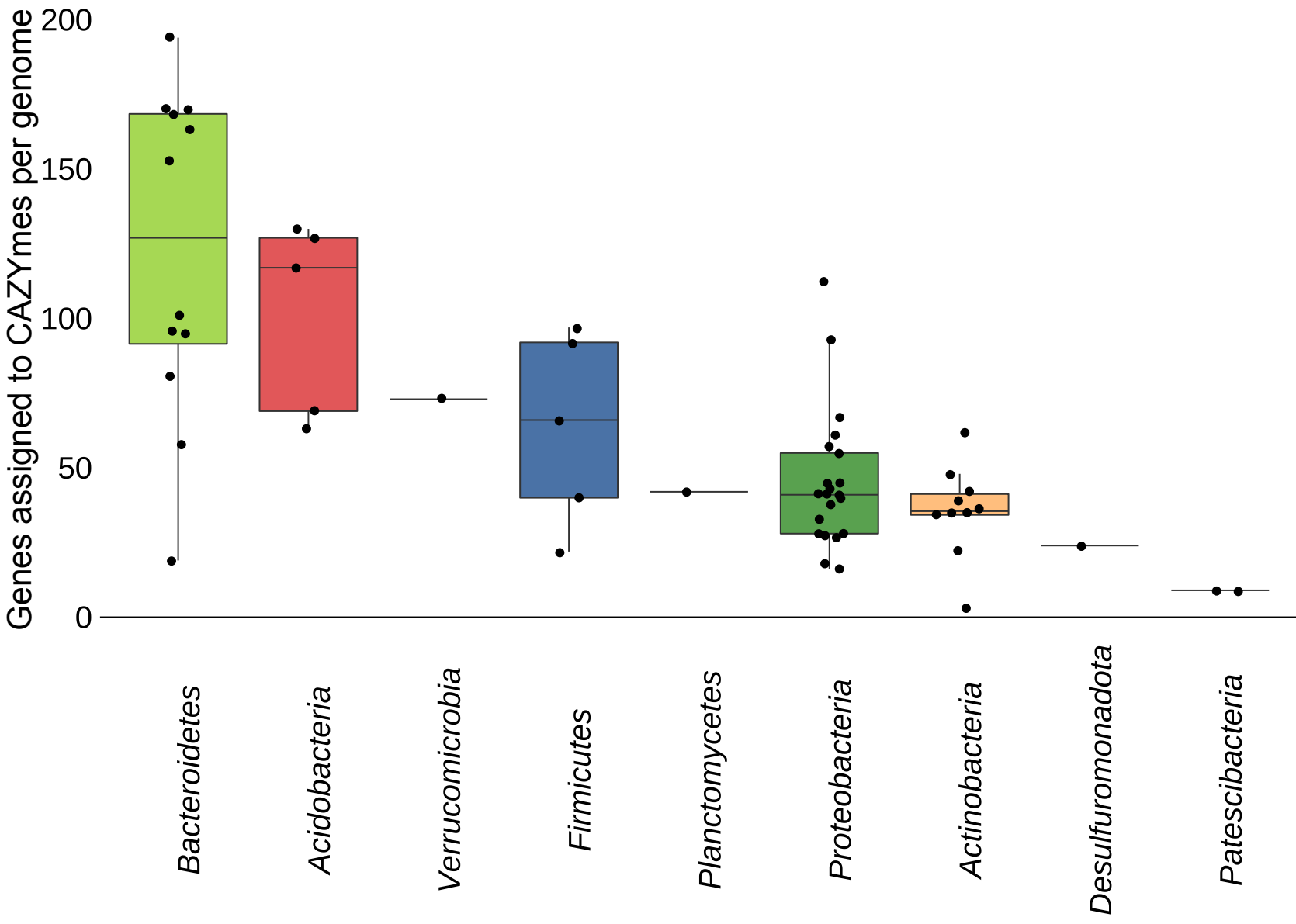

Supplement: FIG S3 [file mSystems.01078-20-sf003.pdf]

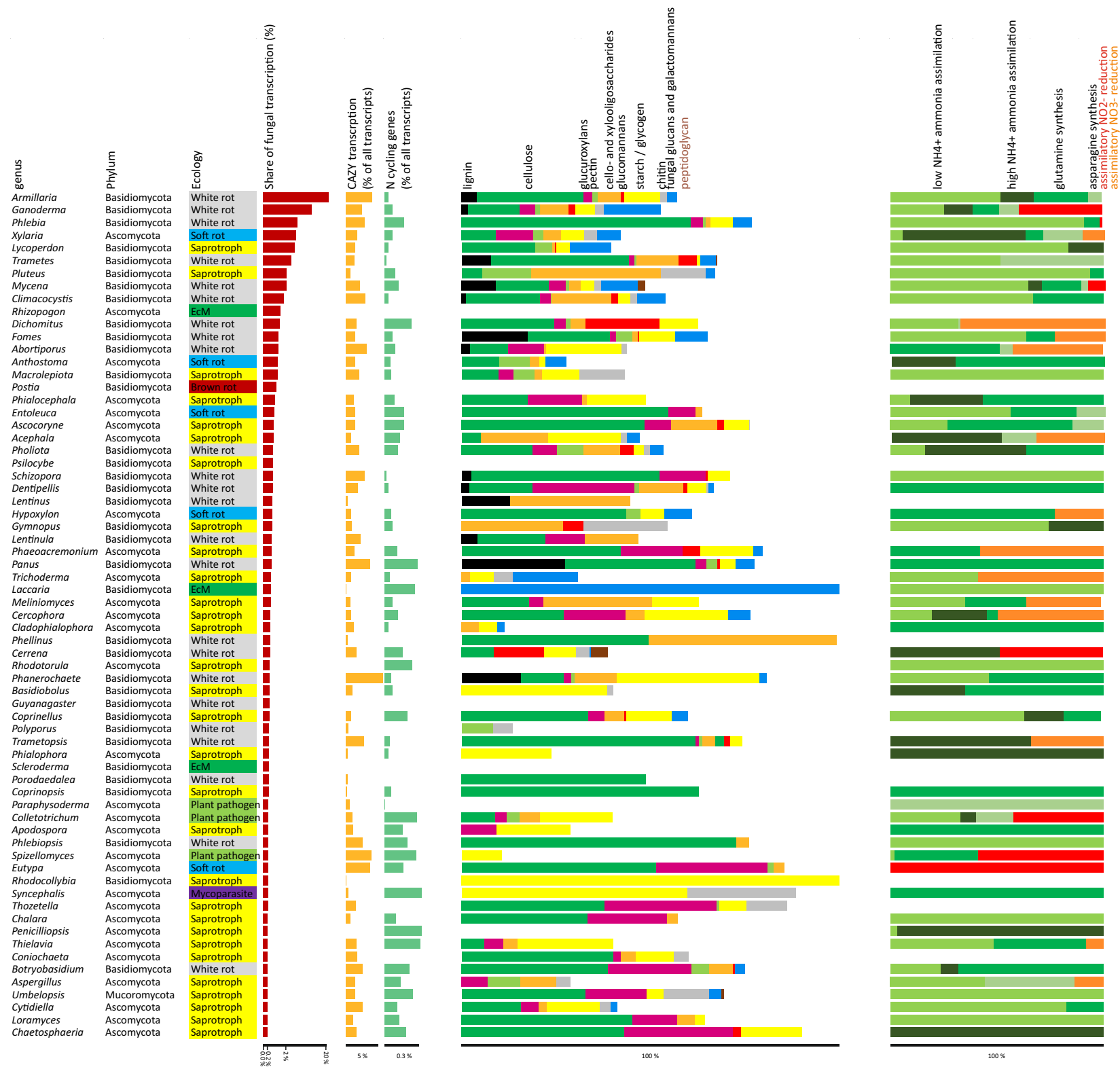

Supplement: FIG S4 [file mSystems.01078-20-sf004.pdf]

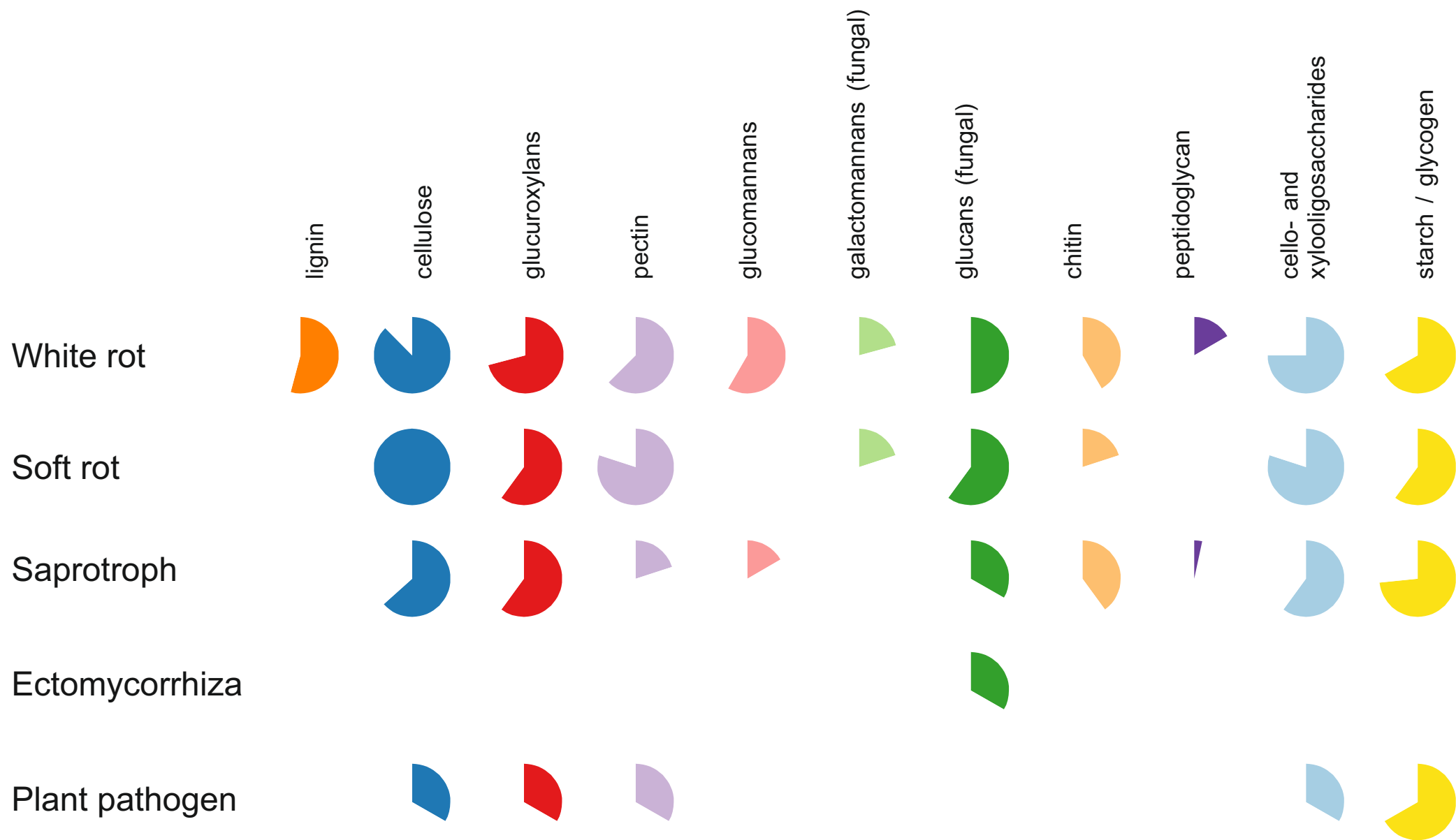

Supplement: FIG S5 [file mSystems.01078-20-sf005.pdf]
