# Supplementary material for: Complementary Roles of Wood-Inhabiting Fungi and Bacteria Facilitate Deadwood Decomposition
Source: mSystems. 2021 Jan 12;6(1):e01078-20. doi: 10.1128/mSystems.01078-20 (PMC7901482; doi:10.1128/mSystems.01078-20)
Supplement: FIG S2 [file mSystems.01078-20-sf002.pdf]

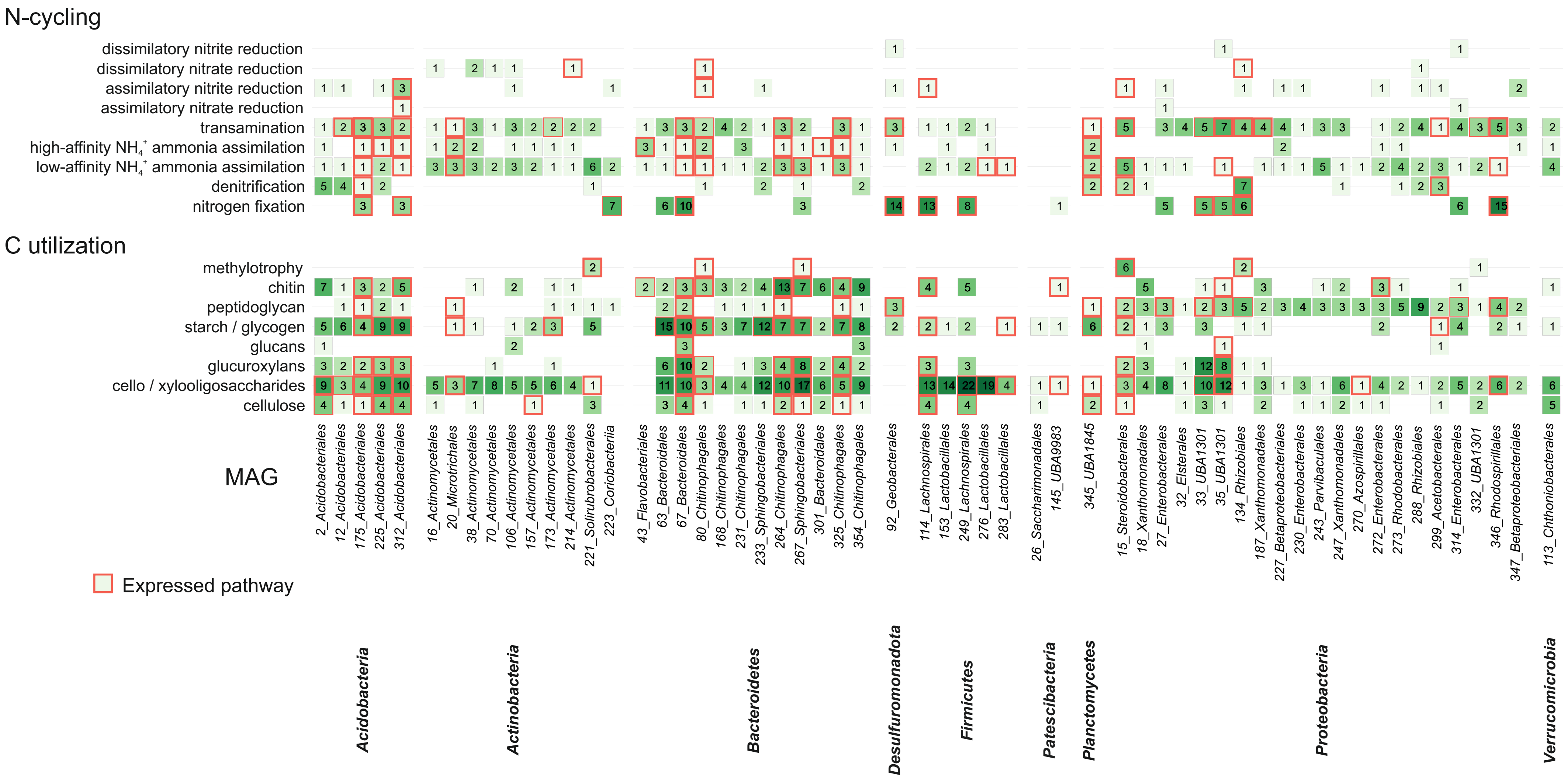
N-cycling
dissimilatory nitrite reduction
dissimilatory nitrate reduction
assimilatory nitrite reduction
assimilatory nitrate reduction
transamination
high-affinity NH4+ ammonia assimilation
low-affinity NH4+ ammonia assimilation
denitrification
nitrogen fixation
C utilization
methylotrophy
chitin
peptidoglycan
starch / glycogen
glucans
glucuroxylans
cello / xylooligosaccharides
cellulose
MAG
2_Acidobacteriales
12_Acidobacteriales
175_Acidobacteriales
225_Acidobacteriales
312_Acidobacteriales
16_Actinomycetales
20_Microtrichales
38_Actinomycetales
70_Actinomycetales
106_Actinomycetales
157_Actinomycetales
173_Actinomycetales
214_Actinomycetales
221_Solirubrobacterales
223_Coriobacteriia
43_Flavobacteriales
63_Bacteroidales
67_Bacteroidales
80_Chitinophagales
168_Chitinophagales
231_Chitinophagales
233_Sphingobacteriales
264_Chitinophagales
267_Sphingobacteriales
301_Bacteroidales
325_Chitinophagales
354_Chitinophagales
92_Geobacterales
114_Lachnospirales
153_Lactobacillales
249_Lachnospirales
276_Lactobacillales
283_Lactobacillales
26_Saccharimonadales
145_UBA9983
345_UBA1845
15_Steroidobacterales
18_Xanthomonadales
27_Enterobacterales
32_Elsterales
33_UBA1301
35_UBA1301
134_Rhizobiales
187_Xanthomonadales
227_Betaproteobacteriales
230_Enterobacterales
243_Parvibaculales
247_Xanthomonadales
270_Azospirillales
272_Enterobacterales
273_Rhodobacterales
288_Rhizobiales
299_Acetobacterales
314_Enterobacterales
332_UBA1301
346_Rhodospirillales
347_Betaproteobacteriales
113_Chthoniobacterales
Expressed pathway
Acidobacteria
Actinobacteria
Bacteroidetes
Desulfuromonadota
Firmicutes
Patescibacteria
Planctomycetes
Proteobacteria
Verrucomicrobia
