# Supplementary material for: Complementary Roles of Wood-Inhabiting Fungi and Bacteria Facilitate Deadwood Decomposition
Source: mSystems. 2021 Jan 12;6(1):e01078-20. doi: 10.1128/mSystems.01078-20 (PMC7901482; doi:10.1128/mSystems.01078-20)
Supplement: TABLE S1 [file mSystems.01078-20-st001.docx]

| **CAZY family** | **main activity** | **target** | **target source** |
| --- | --- | --- | --- |
| AA2 | ligninolytic Class II peroxidases | lignin | plants |
| AA9 | C1-hydroxylating/C4 dehydrogenating (EC 1.14.99.54, EC 1.14.99.56) | cellulose | plants |
| GH12 | endoglucanase (EC 3.2.1.4) | cellulose | plants |
| GH124 | endoglucanase (EC 3.2.1.4) | cellulose | plants |
| GH44 | endoglucanase/endo-β-1,4-mannanase (EC 3.2.1.4,EC 3.2.1.78) | cellulose | plants |
| GH45 | endoglucanase (EC 3.2.1.4) | cellulose | plants |
| GH48 | reducing end-acting cellobiohydrolase/endoglucanase (EC 3.2.1.176,EC 3.2.1.4) | cellulose | plants |
| GH5_1 | endoglucanase (EC 3.2.1.4) | cellulose | plants |
| GH5_2 | endoglucanase (EC 3.2.1.4) | cellulose | plants |
| GH5_25 | endoglucanase (EC 3.2.1.4) | cellulose | plants |
| GH5_26 | endoglucanase (EC 3.2.1.4) | cellulose | plants |
| GH5_37 | endoglucanase (EC 3.2.1.4) | cellulose | plants |
| GH5_38 | endoglucanase (EC 3.2.1.4) | cellulose | plants |
| GH5_39 | endoglucanase (EC 3.2.1.4) | cellulose | plants |
| GH5_4 | endoglucanase (EC 3.2.1.4) | cellulose | plants |
| GH5_46 | endo-β-1,4-mannanase (EC 3.2.1.78) | cellulose | plants |
| GH5_5 | endoglucanase (EC 3.2.1.4) | cellulose | plants |
| GH6 | cellobiohydrolase (EC 3.2.1.91) | cellulose | plants |
| GH7 | reducing end-acting cellobiohydrolase (EC 3.2.1.176) | cellulose | plants |
| GH8 | endoglucanase/exo-β-1,4-glucanase (EC 3.2.1.4, EC 3.2.1.74) | cellulose | plants |
| GH9 | endoglucanase (EC 3.2.1.4) | cellulose | plants |
| CE1 | acetyl xylan esterase (EC 3.1.1.72) | glucuroxylans | plants |
| CE12 | acetyl xylan esterase (EC 3.1.1.72) | glucuroxylans | plants |
| CE15 | acetyl xylan esterase (EC 3.1.1.72) | glucuroxylans | plants |
| CE2 | acetyl xylan esterase (EC 3.1.1.72) | glucuroxylans | plants |
| CE3 | acetyl xylan esterase (EC 3.1.1.72) | glucuroxylans | plants |
| CE4 | acetyl xylan esterase (EC 3.1.1.72) | glucuroxylans | plants |
| CE5 | acetyl xylan esterase (EC 3.1.1.72) | glucuroxylans | plants |
| CE6 | acetyl xylan esterase (EC 3.1.1.72) | glucuroxylans | plants |
| CE7 | acetyl xylan esterase (EC 3.1.1.72) | glucuroxylans | plants |
| GH10 | endo-β-1,4-xylanase (EC 3.2.1.8) | glucuroxylans | plants |
| GH11 | endo-β-1,4-xylanase (EC 3.2.1.8) | glucuroxylans | plants |
| GH115 | xylan alfa-1,2-glucuronidase (EC 3.2.1.131) | glucuroxylans | plants |
| GH141 | endo-β-1,4-xylanase (EC 3.2.1.8) | glucuroxylans | plants |
| GH30_7 | endo-β-1,4-xylanase (EC 3.2.1.8) | glucuroxylans | plants |
| GH30_8 | endo-β-1,4-xylanase (EC 3.2.1.8) | glucuroxylans | plants |
| GH43_16 | endo-β-1,4-xylanase (EC 3.2.1.8) | glucuroxylans | plants |
| GH43_29 | endo-β-1,4-xylanase (EC 3.2.1.8) | glucuroxylans | plants |
| GH5_21 | endo-β-1,4-xylanase (EC 3.2.1.8) | glucuroxylans | plants |
| GH5_35 | endo-β-1,4-xylanase (EC 3.2.1.8) | glucuroxylans | plants |
| GH67 | xylan alfa-1,2-glucuronidase (EC 3.2.1.131) | glucuroxylans | plants |
| CE8 | pectin methylesterase (EC 3.1.1.11) | pectin | plants |
| GH106 | alfa-L-rhamnosidase (EC 3.2.1.40) | pectin | plants |
| GH28 | polygalacturonase (EC 3.2.1.15); alfa-L-rhamnosidase (EC 3.2.1.40); exo-polygalacturonase (EC 3.2.1.67); exo-polygalacturonosidase (EC 3.2.1.82); rhamnogalacturonase (EC 3.2.1.171); rhamnogalacturonan alfa-1,2-galacturonohydrolase (EC 3.2.1.173); endo-xylogalacturonan hydrolase (EC 3.2.1.-) | pectin | plants |
| GH78 | alfa-L-rhamnosidase (EC 3.2.1.40) | pectin | plants |
| PL1_1 | pectate lyase (EC 4.2.2.2) | pectin | plants |
| PL1_2 | pectate lyase (EC 4.2.2.2) | pectin | plants |
| PL1_3 | pectate lyase (EC 4.2.2.2) | pectin | plants |
| PL1_4 | pectin lyase (EC 4.2.2.10) | pectin | plants |
| PL1_5 |  | pectin | plants |
| PL1_6 | pectate lyase (EC 4.2.2.2) | pectin | plants |
| PL1_7 | pectate lyase (EC 4.2.2.2) | pectin | plants |
| PL1_8 | pectin lyase (EC 4.2.2.10) | pectin | plants |
| PL10 | pectate lyase (EC 4.2.2.2) | pectin | plants |
| PL11 |  | pectin | plants |
| PL2_1 | pectate lyase (EC 4.2.2.2) | pectin | plants |
| PL2_2 | exo-pectate lyase (EC 4.2.2.9) | pectin | plants |
| PL3 | pectate lyase (EC 4.2.2.2) | pectin | plants |
| PL4 |  | pectin | plants |
| PL9_1 |  | pectin | plants |
| GH1 | β-glucosidase (EC 3.2.1.21) | cello/xylobiose | plants |
| GH116 | β-glucosidase/β-xylosidase (EC 3.2.1.21,EC 3.2.1.37) | cello/xylobiose | plants |
| GH120 | β-xylosidase (EC 3.2.1.37) | cello/xylobiose | plants |
| GH3 | β-glucosidase (EC 3.2.1.21) | cello/xylobiose | plants |
| GH30_2 | β-xylosidase (EC 3.2.1.37) | cello/xylobiose | plants |
| GH30_6 | β-glucosidase (EC 3.2.1.21) | cello/xylobiose | plants |
| GH39 | β-xylosidase (EC 3.2.1.37) | cello/xylobiose | plants |
| GH43_1 | β-xylosidase (EC 3.2.1.37) | cello/xylobiose | plants |
| GH43_11 | β-xylosidase (EC 3.2.1.37) | cello/xylobiose | plants |
| GH43_14 | β-xylosidase (EC 3.2.1.37) | cello/xylobiose | plants |
| GH43_22 | β-xylosidase (EC 3.2.1.37) | cello/xylobiose | plants |
| GH43_27 | β-xylosidase (EC 3.2.1.37) | cello/xylobiose | plants |
| GH43_35 | β-xylosidase (EC 3.2.1.37) | cello/xylobiose | plants |
| GH5_22 | β-xylosidase (EC 3.2.1.37) | cello/xylobiose | plants |
| GH5_43 | β-glucosidase (EC 3.2.1.21) | cello/xylobiose | plants |
| GH5_45 | β-glucosidase (EC 3.2.1.21) | cello/xylobiose | plants |
| GH52 | β-xylosidase (EC 3.2.1.37) | cello/xylobiose | plants |
| GH113 | endo-β-1,4-mannanase (EC 3.2.1.78) | glucomannans | plants / fungi |
| GH134 | endo-β-1,4-mannanase (EC 3.2.1.78) | glucomannans | plants / fungi |
| GH26 | endo-β-1,4-mannanase (EC 3.2.1.78) | glucomannans | plants / fungi |
| GH5_10 | endo-β-1,4-mannanase (EC 3.2.1.78) | glucomannans | plants / fungi |
| GH5_17 | endo-β-1,4-mannanase (EC 3.2.1.78) | glucomannans | plants / fungi |
| GH5_18 | β-mannosidase (EC 3.2.1.25) | glucomannans | plants / fungi |
| GH5_19 | β-mannosidase (EC 3.2.1.25) | glucomannans | plants / fungi |
| GH5_36 | endo-β-1,4-mannanase (EC 3.2.1.78) | glucomannans | plants / fungi |
| GH5_40 | endo-β-1,4-mannanase (EC 3.2.1.78) | glucomannans | plants / fungi |
| GH5_41 | endo-β-1,4-mannanase (EC 3.2.1.78) | glucomannans | plants / fungi |
| GH5_55 | endo-β-1,4-mannanase (EC 3.2.1.78) | glucomannans | plants / fungi |
| GH5_7 | endo-β-1,4-mannanase (EC 3.2.1.78) | glucomannans | plants / fungi |
| GH5_8 | endo-β-1,4-mannanase (EC 3.2.1.78) | glucomannans | plants / fungi |
| GH119 | alfa-amylase (EC 3.2.1.1) | starch/glycogen | plants / fungi |
| GH122 | alfa-glucosidase (EC 3.2.1.20) | starch/glycogen | plants / fungi |
| GH13 | alfa-amylase/alfa-glucosidase/amylopullulanase (EC 3.2.1.1,EC 3.2.1.20,EC 3.2.1.41) | starch/glycogen | plants / fungi |
| GH133 | amylo-alfa-1,6-glucosidase (EC 3.2.1.33) | starch/glycogen | plants / fungi |
| GH14 | β-amylase (EC 3.2.1.2) | starch/glycogen | plants / fungi |
| GH15 | glucoamylase (EC 3.2.1.3) | starch/glycogen | plants / fungi |
| GH31 | alfa-glucosidase (EC 3.2.1.20) | starch/glycogen | plants / fungi |
| GH57 | alfa-amylase/amylopullulanase (EC 3.2.1.1,EC 3.2.1.41) | starch/glycogen | plants / fungi |
| GH77 | amylomaltase or 4-alfa-glucanotransferase (EC 2.4.1.25) | starch/glycogen | plants / fungi |
| AA10 | lytic chitin monooxygenase/C1-hydroxylating/C4 dehydrogenating (EC 1.14.99.53,EC 1.14.99.54,EC 1.14.99.56) | chitin | fungi |
| AA11 | lytic chitin monooxygenase (EC 1.14.99.53) | chitin | fungi |
| GH18 | chitinase (EC 3.2.1.14) | chitin | fungi |
| GH19 | chitinase (EC 3.2.1.14) | chitin | fungi |
| GH20 | N-acetyl β-glucosaminidase (EC 3.2.1.52) | chitin | fungi |
| GH128 | endo-1,3-β-glucanase (EC 3.2.1.39) | glucans | fungi |
| GH152 | endo-1,3-β-glucanase (EC 3.2.1.39) | glucans | fungi |
| GH157 | endo-1,3-β-glucanase (EC 3.2.1.39) | glucans | fungi |
| GH158 | endo-1,3-β-glucanase (EC 3.2.1.39) | glucans | fungi |
| GH17 | endo-1,3-β-glucanase (EC 3.2.1.39) | glucans | fungi |
| GH30_3 | endo-1,6-β-glucosidase (EC 3.2.1.75) | glucans | fungi |
| GH5_14 | endo-1,3-β-glucanase/β-glucosidase (EC 3.2.1.39,EC 3.2.1.21) | glucans | fungi |
| GH5_15 | endo-1,6-β-glucosidase (EC 3.2.1.75) | glucans | fungi |
| GH5_9 | exo-β-1,3-glucanase (EC 3.2.1.58) | glucans | fungi |
| GH64 | endo-1,3-β-glucanase (EC 3.2.1.39) | glucans | fungi |
| GH71 | alfa-1,3-glucanase (EC 3.2.1.59) | glucans | fungi |
| GH81 | endo-1,3-β-glucanase (EC 3.2.1.39) | glucans | fungi |
| GH87 | alfa-1,3-glucanase (EC 3.2.1.59) | glucans | fungi |
| GH110 | alfa-galactosidase (EC 3.2.1.22) | galactomannans | fungi |
| GH27 | alfa-galactosidase (EC 3.2.1.22) | galactomannans | fungi |
| GH36 | alfa-galactosidase (EC 3.2.1.22) | galactomannans | fungi |
| GH97 | alfa-galactosidase (EC 3.2.1.22) | galactomannans | fungi |
| GH102 | peptidoglycan lytic transglycosylase (EC 4.2.2.n1) | peptidoglycan | bacteria |
| GH103 | peptidoglycan lytic transglycosylase (EC 4.2.2.n1) | peptidoglycan | bacteria |
| GH104 | peptidoglycan lytic transglycosylase (EC 4.2.2.n1) | peptidoglycan | bacteria |
| GH108 | lysozyme (EC 3.2.1.17) | peptidoglycan | bacteria |
| GH22 | lysozyme (EC 3.2.1.17) | peptidoglycan | bacteria |
| GH23 | lysozyme/peptidoglycan lytic transglycosylase (EC 3.2.1.17,EC 4.2.2.n1) | peptidoglycan | bacteria |
| GH24 | lysozyme (EC 3.2.1.17) | peptidoglycan | bacteria |
| GH25 | lysozyme (EC 3.2.1.17) | peptidoglycan | bacteria |
