# Supplementary material for: Complementary Roles of Wood-Inhabiting Fungi and Bacteria Facilitate Deadwood Decomposition
Source: mSystems. 2021 Jan 12;6(1):e01078-20. doi: 10.1128/mSystems.01078-20 (PMC7901482; doi:10.1128/mSystems.01078-20)
Supplement: TABLE S2 [file mSystems.01078-20-st002.docx]

| **KEGG class** | **gene** | **function** |
| --- | --- | --- |
| [K16157] | mmoX | methane monooxygenase component A alpha chain [EC:1.14.13.25] |
| [K16158] | mmoY | methane monooxygenase component A beta chain [EC:1.14.13.25] |
| [K16159] | mmoZ | methane monooxygenase component A gamma chain [EC:1.14.13.25] |
| [K16160] | mmoB | methane monooxygenase regulatory protein B |
| [K16161] | mmoC | methane monooxygenase component C [EC:1.14.13.25] |
| [K16162] | mmoD | methane monooxygenase component D [EC:1.14.13.25] |
| [K17066] | MOX | alcohol oxidase [EC:1.1.3.13] |
| [K10944] | pmoA-amoA | methane/ammonia monooxygenase subunit A [EC:1.14.18.3 1.14.99.39] |
| [K10945] | pmoB-amoB | methane/ammonia monooxygenase subunit B |
| [K10946] | pmoC-amoC | methane/ammonia monooxygenase subunit C |
| [K14028] | mdh1/mxaF | methanol dehydrogenase (cytochrome c) subunit 1 [EC:1.1.2.7] |
| [K14029] | mdh2/mxaI | methanol dehydrogenase (cytochrome c) subunit 2 [EC:1.1.2.7] |
