# Supplementary material for: Complementary Roles of Wood-Inhabiting Fungi and Bacteria Facilitate Deadwood Decomposition
Source: mSystems. 2021 Jan 12;6(1):e01078-20. doi: 10.1128/mSystems.01078-20 (PMC7901482; doi:10.1128/mSystems.01078-20)
Supplement: TABLE S3 [file mSystems.01078-20-st003.docx]

| **KEGG class** | **gene** | **function** | **protein** | **process** |
| --- | --- | --- | --- | --- |
| [K00260] | gudB,rocG | glutamate dehydrogenase [EC:1.4.1.2] | glutamate dehydrogenase | high NH4+ ammonia assimilation |
| [K00261] | GLUD1_2,gdhA | glutamate dehydrogenase (NAD(P)+) [EC:1.4.1.3] | glutamate dehydrogenase | high NH4+ ammonia assimilation |
| [K00262] | gdhA | glutamate dehydrogenase (NADP+) [EC:1.4.1.4] | glutamate dehydrogenase | high NH4+ ammonia assimilation |
| [K00264] | GLT1 | glutamate synthase (NADH) [EC:1.4.1.14] | glutamate synthase | transamination |
| [K00265] | gltB | glutamate synthase (NADPH) large chain [EC:1.4.1.13] | glutamate synthase | transamination |
| [K00266] | gltD | glutamate synthase (NADPH) small chain [EC:1.4.1.13] | glutamate synthase | transamination |
| [K00360] | nasB | assimilatory nitrate reductase electron transfer subunit [EC:1.7.99.-] | nitrate reductase | assimilatory nitrate red. |
| [K00362] | nirB | nitrite reductase (NADH) large subunit [EC:1.7.1.15] | nitrite reductase | assimilatory nitrite red. |
| [K00363] | nirD | nitrite reductase (NADH) small subunit [EC:1.7.1.15] | nitrite reductase | assimilatory nitrite red. |
| [K00366] | nirA | ferredoxin-nitrite reductase [EC:1.7.7.1] | nitrite reductase | assimilatory nitrite red. |
| [K00367] | narB | ferredoxin-nitrate reductase [EC:1.7.7.2] | nitrate reductase | assimilatory nitrate red. |
| [K00368] | nirK | nitrite reductase (NO-forming) [EC:1.7.2.1] | nitrite reductase | denitrification |
| [K00370] | narG,narZ,nxrA | nitrate reductase / nitrite oxidoreductase, alpha subunit [EC:1.7.5.1 1.7.99.-] | nitrate reductase | denitrification, dissimilatory nitrate reduction |
| [K00370] | narG,narZ,nxrA | nitrate reductase / nitrite oxidoreductase, alpha subunit [EC:1.7.5.1 1.7.99.-] | nitrate reductase | denitrification, dissimilatory nitrate reduction |
| [K00371] | narH,narY,nxrB | nitrate reductase / nitrite oxidoreductase, beta subunit [EC:1.7.5.1 1.7.99.-] | nitrate reductase | denitrification, dissimilatory nitrate reduction |
| [K00371] | narH,narY,nxrB | nitrate reductase / nitrite oxidoreductase, beta subunit [EC:1.7.5.1 1.7.99.-] | nitrate reductase | denitrification, dissimilatory nitrate reduction |
| [K00372] | nasA | assimilatory nitrate reductase catalytic subunit [EC:1.7.99.-] | nitrate reductase | dissimilatory nitrate red. |
| [K00373] | narJ, narW | nitrate reductase molybdenum cofactor assembly chaperone NarJ/NarW | nitrate reductase | dissimilatory nitrate red. |
| [K00374] | narI,narV | nitrate reductase gamma subunit [EC:1.7.5.1 1.7.99.-] | nitrate reductase | denitrification, dissimilatory nitrate reduction |
| [K00374] | narI,narV | nitrate reductase gamma subunit [EC:1.7.5.1 1.7.99.-] | nitrate reductase | denitrification, dissimilatory nitrate reduction |
| [K00376] | nosZ | nitrous-oxide reductase [EC:1.7.2.4] | nitrous oxide reductase | denitrification |
| [K00531] | anfG | nitrogenase delta subunit [EC:1.18.6.1] | nitrogenase | nitrogen fixation |
| [K01915] | glnA,GLUL | glutamine synthetase [EC:6.3.1.2] | glutamine synthetase | low NH4+ ammonia assimilation |
| [K02305] | norC | nitric oxide reductase subunit C | nitric oxide reductase | denitrification |
| [K02567] | napA | periplasmic nitrate reductase NapA [EC:1.7.99.-] | nitrate reductase | denitrification, dissimilatory nitrate reduction |
| [K02567] | napA | periplasmic nitrate reductase NapA [EC:1.7.99.-] | nitrate reductase | denitrification, dissimilatory nitrate reduction |
| [K02568] | napB | cytochrome c-type protein NapB | nitrate reductase | denitrification, dissimilatory nitrate reduction |
| [K02568] | napB | cytochrome c-type protein NapB | nitrate reductase | denitrification, dissimilatory nitrate reduction |
| [K02586] | nifD | nitrogenase molybdenum-iron protein alpha chain [EC:1.18.6.1] | nitrogenase | nitrogen fixation |
| [K02588] | nifH | nitrogenase iron protein NifH | nitrogenase | nitrogen fixation |
| [K02591] | nifK | nitrogenase molybdenum-iron protein beta chain [EC:1.18.6.1] | nitrogenase | nitrogen fixation |
| [K02595] | nifW | nitrogenase-stabilizing/protective protein | nitrogenase | nitrogen fixation |
| [K03385] | nrfA | nitrite reductase (cytochrome c-552) [EC:1.7.2.2] | nitrite reductase | dissimilatory nitrite red. |
| [K04013] | nrfB | cytochrome c-type protein NrfB | nitrite reductase | dissimilatory nitrite red. |
| [K04014] | nrfC | protein NrfC | nitrite reductase | dissimilatory nitrite red. |
| [K04015] | nrfD | protein NrfD | nitrite reductase | dissimilatory nitrite red. |
| [K04561] | norB | nitric oxide reductase subunit B [EC:1.7.2.5] | nitric oxide reductase | denitrification |
| [K10534] | NR | nitrate reductase (NAD(P)H) [EC:1.7.1.1 1.7.1.2 1.7.1.3] | nitrate reductase | assimilatory nitrate red. |
| [K10535] | hao | hydroxylamine dehydrogenase [EC:1.7.2.6] | hydroxylamine dehydrogenase | nitrification |
| [K10944] | pmoA-amoA | methane/ammonia monooxygenase subunit A [EC:1.14.18.3 1.14.99.39] | methane/ammonia monooxygenase | nitrification |
| [K10945] | pmoB-amoB | methane/ammonia monooxygenase subunit B | methane/ammonia monooxygenase | nitrification |
| [K10946] | pmoC-amoC | methane/ammonia monooxygenase subunit C | methane/ammonia monooxygenase | nitrification |
| [K15864] | nirS | nitrite reductase (NO-forming) / hydroxylamine reductase [EC:1.7.2.1 1.7.99.1] | nitrite reductase | denitrification |
| [K15876] | nrfH | cytochrome c nitrite reductase small subunit | nitrite reductase | dissimilatory nitrite red. |
| [K15877] | CYP55 | fungal nitric oxide reductase [EC:1.7.1.14] | nitric oxide reductase | denitrification |
| [K15878] | narB | rieske iron-sulfur protein | protein | dissimilatory nitrate red. |
| [K15879] | narC | cytochrome b-561 | protein | dissimilatory nitrate red. |
| [K17877] | NIT-6 | nitrite reductase (NAD(P)H) [EC:1.7.1.4] | nitrite reductase | assimilatory nitrite red. |
| [K22896] | vnfD | vanadium-dependent nitrogenase alpha chain [EC:1.18.6.2] | nitrogenase | nitrogen fixation |
| [K22897] | vnfK | vanadium-dependent nitrogenase beta chain [EC:1.18.6.2] | nitrogenase | nitrogen fixation |
| [K22898] | vnfG | vanadium nitrogenase delta subunit [EC:1.18.6.2] | nitrogenase | nitrogen fixation |
| [K22899] | vnfH | vanadium nitrogenase iron protein | nitrogenase | nitrogen fixation |
