# Supplementary material for: Complementary Roles of Wood-Inhabiting Fungi and Bacteria Facilitate Deadwood Decomposition
Source: mSystems. 2021 Jan 12;6(1):e01078-20. doi: 10.1128/mSystems.01078-20 (PMC7901482; doi:10.1128/mSystems.01078-20)
Supplement: TABLE S4 [file mSystems.01078-20-st004.docx]

Table S4.: Properties dead *Faqus sylvatica* logs.

| sample ID | location | age class | density  (kg m^-3^) | C content (%) | N content (%) | lignin  (%) | pH | bacterial 16S (millions of copies g^-1^) | fungal 18S (millions of copies g^-1^) | F/B ratio | respiration  (mg CO_2_ kg^-1^ h^-1^) | N_2_ fixation  (µg kg^-1^ d^-1^) |
| --- | --- | --- | --- | --- | --- | --- | --- | --- | --- | --- | --- | --- |
| 6 | 48.666377 N 14.709879 E | young | 382.0 | 50.7 | 0.71 | 40.4 | 3.95 | 1312 | 1508 | 1.15 | 77.0 | 411.4 |
| 7 | 48.666310 N 14.709489 E | young | 292.7 | 22.5 | 0.12 | 26.3 | 4.76 | 2482 | 1044 | 0.42 | 49.5 | 184.5 |
| 44 | 48.665969 N 14.706625 E | young | 470.2 | 50.3 | 0.43 | 30.4 | 3.87 | 1564 | 3387 | 2.17 | 65.0 | 252.6 |
| 110 | 48.665321 N 14.708803 E | young | 417.4 | 49.6 | 0.68 | 12.5 | 4.12 | 855 | 2429 | 2.84 | 41.4 | 15.3 |
| 116 | 48.666959 N 14.704826 E | young | 420.7 | 51.3 | 0.69 | 34.3 | 3.32 | 664 | 1877 | 2.83 | 64.1 | 622.5 |
| 31 | 48.666891 N 14.703543 E | old | 412.9 | 48.7 | 0.34 | 20.2 | 4.87 | 2458 | 1349 | 0.55 | 35.6 | 7.9 |
| 49 | 48.665517 N 14.706880 E | old | 304.3 | 49.3 | 0.23 | 26.6 | 4.44 | 840 | 1458 | 1.80 | 45.6 | 19.7 |
| 55 | 48.665975 N 14.709224 E | old | 427.3 | 49.9 | 0.33 | 38.8 | 3.99 | 643 | 2841 | 4.42 | 69.5 | 26.9 |
| 69 | 48.664308 N 14.704474 E | old | 332.1 | 48.2 | 0.50 | 24.3 | 4.78 | 973 | 144 | 0.15 | 23.6 | 14.4 |
| 106 | 48.664703 N 14.708726 E | old | 400.8 | 49.2 | 0.40 | 22.8 | 4.07 | 1139 | 957 | 0.84 | 77.3 | 113.6 |
